# Impact of Health Counselling on Cardiovascular Disease Risk in Middle Aged Men: Influence of Socioeconomic Status

**DOI:** 10.1371/journal.pone.0088959

**Published:** 2014-02-14

**Authors:** Reijo Siren, Johan G. Eriksson, Markku Peltonen, Hannu Vanhanen

**Affiliations:** 1 Health Centre of City of Helsinki, Helsinki, Finland; 2 National Institute for Health and Welfare, Helsinki, Finland; 3 Department of General Practice and Primary Health Care, University of Helsinki, Helsinki, Finland; 4 Helsinki University Central Hospital, Unit of General Practice, Helsinki, Finland; 5 Folkhalsan Research Centre, Helsinki, Finland; 6 The Social Insurance Institution of Finland (KELA), Helsinki, Finland; The National Institute for Health Innovation, New Zealand

## Abstract

**Background:**

The inverse association between socioeconomic status and cardiovascular disease is well documented. We examined whether the impact of health counselling on cardiovascular risk factors in middle-aged men differed according to socioeconomic status.

**Methods:**

We used data from a community based study assessing the risk for cardiovascular disease among middle-aged men in Helsinki, Finland. Traditional cardiovascular disease risk factors were measured and cardiovascular disease risk was assessed by a modified risk tool used in the North Karelia project (CVD Risk Score). Those men with increased risk for cardiovascular disease at their baseline visit in 2006 received lifestyle counselling. After two years these high-risk men were invited to a follow-up visit. The same measurements and risk assessments were repeated.

**Results:**

Based on the CVD Risk Score there were significant differences between the groups at baseline (p = 0.001) and at follow-up (p<0.001) with the highest scores in the lowest educational group. There were no significant differences in traditional cardiovascular risk factors according to educational attainment between groups either at baseline or at follow-up. Baseline lifestyle characteristics differed between the groups regarding use of soft fat (p = 0.019). All groups responded positively to lifestyle counselling.

**Conclusions:**

The present study showed that lifestyle counselling is feasible in high-risk middle-aged men and lifestyle intervention works in all educational groups. Interestingly the traditional risk factors did not show improvement, but the risk score improved. From a practical point of view our findings stress the importance of using risk score calculators in health counselling instead of looking at individual cardiovascular disease risk factors.

## Introduction

Cardiovascular disease (CVD) is the major cause of deaths globally. According to the World Health Organization an estimated 17.3 million people died from CVDs in 2008, representing 31% of all global deaths [1]. In Finland among men aged 15–64 years coronary heart disease (CHD) is the second most common cause of deaths, accounting for 15.5% of total mortality in this age group in 2010 [2]. The risk factors for CVD are well established, the main risk factors being smoking, hypertension, dyslipidemia, diabetes, physical inactivity, and obesity. These risk factors are potentially modifiable by health counselling. Several epidemiological studies have shown an inverse association between socioeconomic status (SES) and morbidity and mortality from CVD [3], [4], [5] Socioeconomic status can be classified in different ways, including occupational status, income and educational attainment. Educational attainment is the recommended measure of SES if only one SES measure is available when predicting health outcomes [6], [7] The aim of this study was to evaluate the impact of health counselling based upon personal risk estimation of CVD during a two-year follow-up taking into account socioeconomic status.

## Methods

### Subjects and Study Design

All men aged 40 years living in Helsinki were invited in 2006 to a cardiovascular disease risk evaluation and health-counselling visit at their local health care centre. Of the 4274 men that were invited, 1454 (34%) participated. In the appointment with a trained nurse the participants were interviewed about their lifestyle. CVD risk assessment was made by a modified version of the North Karelia project risk tool [8]. 471 men with a CVD risk score of ≥4.5 received lifestyle counselling based upon their own individual risk profile in accordance with the national guidelines for preventing CVD, which again are based upon European guidelines on cardiovascular disease prevention in clinical practice [9]. A follow-up study protocol for these men with increased risk was designed. Approval of the protocol was obtained from the Helsinki University Central Hospital Ethics Committee. In 2008 a total of 430 of the high risk men were identified, from the local health care centre register, and were invited to a follow-up visit. Of these, 200 men participated and gave written informed consent. At the follow-up visit the same risk assessments and measurements were done with the same techniques as at the baseline visit in 2006. Educational attainment was available for 185 (93%) of the men. We categorised educational attainment into three levels based upon duration of formal schooling: low (≤9 years, n = 31; 16.8%), middle (10 to 12 years, n = 87; 47%), and high (≥13 years, n = 67; 36.2%). The distribution of educational attainment corresponds with the general distribution of educational attainment in this age-group in Finland, being 18.3%, 48.4%, and 33.3% respectively [10].

### CVD Risk Assessment

The CVD Risk Score; a modified version of the North Karelia project risk tool is based on BMI, smoking, physical activity, systolic and diastolic blood pressure, and total cholesterol concentration. Depending of risk factor status a subject can have a risk score from zero to sixteen. A subject with at least 4.5 points is categorized as being at high risk [11]. We also assessed CVD risk by a risk formula derived from Framingham study participants, “General Cardiovascular Risk Profile for Use in Primary care” [12]. The later takes into account gender, age, high density cholesterol, total cholesterol, systolic blood pressure, smoking, and diabetes (the alternative office-based model use BMI instead of total and high density cholesterol). The “Hearth age” definition is based on this risk formula.

### Measurements

Blood pressure measurements were obtained with the subject in the sitting position using an automated sphygmomanometer (Omron HEM-7051-E, Kyoto, Japan). The mean of two measurements was used. Height was measured without shoes to the nearest 0.1 cm, and weight was measured in light indoor clothing to nearest 0.1 kg; BMI was calculated as kg/m^2^. Waist circumference was measured in a standing position, midway between the lowest rib and the iliac crest. All measurements were made according to a standard technique. Blood samples were drawn after an overnight fast by a trained technician and analyzed for lipids and glucose in a certified central laboratory.

### Statistics

*Clinical characteristics and Risk scores*; we used ANOVA analysis of means between groups except for changes between groups we used Univariate General Linear model. When Anova F statistic indicated that there were significant differences between the means, we used Bonferroni correction for multiple group comparisons. Within group analysis we performed with paired samples t-test. The above analysis was made by PASW/SPSS software.

*Lifestyle characteristics*; analysis for proportions between groups we made with Freeman-Halton extension of Fisher’s exact test at: www.vassarstats.net/fisher2×3.html. Changes between groups analyzed with Binary Logistic Regression model of PASW/SPSS software. Within group analysis we performed with Wilson’s method for paired samples proportions and their differences using Confidence Intervals Analysis (CIA) software for Windows. We had PASW statistics for Windows, version 18.0. (SPSS INC., Chicago IL, USA).

## Results

The clinical characteristics of the study population are shown in Table 1. There were no significant differences in the clinical characteristics, between the groups either at baseline or at follow-up visits, when grouped according to educational attainment. Likewise, changes in the parameters assessed did not differ significantly between the groups with the exception of TG concentration (p = 0.05). Significant within the groups changes were observed between baseline and follow-up for HDL cholesterol and glucose concentrations; the former was lower at follow-up in all groups and the latter was higher at the follow-up visit in the two highest educational attainment groups.

**Table 1:** Clinical characteristic at baseline and two years follow-up.

	Educational attainment					
	Low	Middle	High	P value ^a^	All participants	P value ^b^
	n = 31	n = 87	n = 67		n = 200	
BMI (kg/m^2^)						
Baseline	29.9 (3.8)	29.3 (4.7)	29.2 (5.8)	0.804	29.4 (5.0)	
Follow-up	30.2 (4.3)	29.7 (5.2)	29.0 (5.5)	0.482	29.6 (5.2)	
Change	0.3(1.8)	0.4 (2.1)	−0.2(1.5)	0.187	0.2 (1.9)	0.183
95% CI	−0.3 to 1.0	−0.1 to 0.8	−0.5 to 0.2		−0.1 to 0.4	
WC (cm)						
Baseline	105.2(11.8)	102.0 (11.2)	101.4 (12.8)	0.371	102.7 (11.9)	
Follow-up	105.0 (12.3)	103.0 (11.7)	100.5 (13.7)	0.171	102.9 (12.7)	
Change	−0.2 (0.38)	1.0 (5.6)	−0.9 (5.3)	0.123	0.2 (5.3)	0.591
95% CI	−1.7 to 1.4	−0.3 to 2.3	−2.2 to 0.5		−0.6 to 1.0	
SBP (mmHg)						
Baseline	141.9 (21.5)	136.7 (16.5)	137.4 (12.9)	0.296	137.8 (16.0)	
Follow-up	141.2 (14.7)	138.2 (16.4)	137.4 (15.2)	0.583	138.4 (15.5)	
Change	−0.7 (19.7)	1.5 (15.2)	0.0 (15.0)	0.823	0.6 (15.5)	0.578
95% CI	−8.0 to 6.5	−1.7 to 4.8	−3.7 to 3.7		−1.6 to 2.8	
DBP (mmHg)						
Baseline	92.6 (13.0)	90.7 (11.5)	91.3 (10.2)	0.741	91.1 (11.1)	
Follow-up	90.2 (8.9)	90.0 (11.0)	90.0 (10.2)	0.996	90.1 (10.2)	
Change	−2.4 (12.8)	−0.7 (11.4)	−1.3 (10.5)	0.954	−1.0 (11.0)	0.198
95% CI	−7.0 to 2.3	−3.1 to 1.7	−3.9 to 1.3		−2.7 to 0.5	
Total-C (mmol/l)						
Baseline	5.6 (0.97)	5.6 (1.0)	5.7 (1.0)	0.802	5.6 (1.0)	
Follow-up	5.4 (0.96)	5.4 (1.0)	5.5 (0.9)	0.830	5.4 (0.9)	
Change	−0.2 (1.03)	−0.2 (0.9)	−0.2 (0.9)	0.982	−0.2 (0.9)	0.003
95% CI	−0.6 to 0.2	−0.4 to 0.0	−0.4 to 0.0		−0.3 to −0.1	
LDL-C (mmol/l)						
Baseline	3.22 (1.00)	3.35 (0.91)	3.38 (0.91)	0.661	3.33 (0.91)	
Follow-up	3.08 (0.63)	3.12 (0.78)	3.29 (0.86)	0.340	3.16 (0.78))	
Change	−0.14 (0.92)	−0.23 (0.69)	−0.09 (0.69)	0.346	−0.17 (0.72)	0.002
95% CI	−0.49 to 0.22	−0.38 to 0.08	−0.27 to 0.08		−0.27 to −0.06	
HDL-C (mmol/l)						
Baseline	1.48 (0.46)	1.48 (0.37)	1.43 (0.27)	0.811	1.48 (0.35)	
Follow-up	1.22 (0.34)	1.30 (0.32)	1.28 (0.27)	0.367	1.29 (0.33)	
Change	−0.26 (0.34)	−0.18 (0.22)	−0.15 (0.18)	0.058	−0.18 (0.24)	<0.001
95% CI	−0.39 to −0.14	−0.23 to −0.14	−0.19 to −0.11		−0.22 to −0.15	
TG (mmol/l)						
Baseline	2.29 (1.20)	1.89 (1.65)	2.01 (1.39)	0.621	2.01 (1.52)	
Follow-up	2.39 (1.79)	1.98 (1.59)	1.75 (0.75)	0.104	1.98 (1.39)	
Change	0.17 (1.29)	0.09 (1.12)	−0.26 (1.18)	0.050	−0.03 (1.18)	0.130
95% CI	−0.30 to 0.65	−0.15 to 0.34	−0.55 to 0.03		−0.20 to 0.13	
Glucose (mmol/l)						
Baseline	5.6 (1.0)	5.5 (0.5)	5.5 (0.6)	0.524	5.6 (0.6)	
Follow-up	5.8 (0.5)	5.7 (0.6)	5.8 (0.7)	0.743	5.8 (0.6)	
Change	0.2 (0.4)	0.2 (0.5)	0.3 (0.6)	0.578	0.2 (0.5)	<0.001
95% CI	−0.0 to 0.3	0.1 to 0.3	0.1 to 0.4		0.1 to 0.3	

Data are means (SD), ^a^ P values are for test of equality between educational groups, ^b^ P values are test for paired difference. BMI = body mass index, WC = waist circumference, SBP = systolic blood pressure, DBP = diastolic blood pressure, Total-C = total cholesterol, LDL-C = low density cholesterol, HDL-C = high density cholesterol, TG = triglycerides.

Lifestyle characteristics and effects of the intervention on lifestyle are shown in Table 2. At baseline there was a significant difference between groups regarding the use of unsaturated fat; those belonging to the lowest educational group used less unsaturated fat than those in the other two groups. The other lifestyle characteristics did not differ significantly between the groups either at baseline or at follow-up. The desired effect of the counselling was observed in three assessed lifestyle characteristics in all groups. In the highest educational group smoking prevalence decreased most, physical activity increased most in the middle group and replacement of saturated fats with unsaturated fats was most frequent in the low education group.

**Table 2:** Lifestyle characteristics at baseline and at 2 years follow-up.

	Educational attainment					
	Low	Middle	High	P value^a^	All participants	P value^b^
	n = 31	n = 87	n = 67		n = 200	
Smoking %						
Baseline	71.0	59.8	50.7	0.170	59.5	
Follow-up	64.5	54.0	41.8	0.092	52.0	
Change	−6.5	−5.8	−8.9	0.486	−7.5	0.003
95% CI	−20.1 to 7.4	−13.8 to 2.5	−16.1 to −1.6		−12.3 to −2.6	
PA ≥90 min/wk %						
Baseline	9.7	10.3	17.9	0.357	13.5	
Follow-up	25.8	27.6	29.4	0.928	27.0	
Change	16.1	17.3	11.5	0.945	13.5	<0.001
95% CI	−0.15 to 33.6	8.4 to 26.5	0.9 to 22.9		7.6 to 19.5	
Users of soft fat %						
Baseline	3.4	23.5	12.1	0.019	18.0	
Follow-up	46.7	56.5	46.3	0.396	51.5	
Change	43.3	33.0	34.2	0.827	33.5	<0.001
95% CI	22.3 to 60.7	22.2 to 42.4	21.1 to 44.6		26.4 to 39.8	

PA = physical activity,

a P values are for test of equality between educational groups,

b P values are test for paired difference.

Risk scores are shown in Table 3. There were significant differences between the educational groups at baseline in the CVD score (F = 7.395, p = 0.001). The differences between the low and middle educational groups and between the low and high educational groups were significant (p = 0.040 and p = 0.001), respectively. However between the middle and high educational groups the difference was not significant (p = 0.173). Likewise, at follow-up there were significant differences between the educational groups (F 0 8.218, p<0.001). After Bonferroni correction the difference between the low and middle educational groups was not significant (p = 0.117) while that between the low and high educational groups and between the middle and high educational groups the differences remained statistically significant (p<0.001 and p = 0.033), respectively. According to the Framingham risk score the differences between the educational groups were significant only at the follow-up visits (F = 4.132, p = 0.018). Between the low and middle educational groups and between low and high educational groups the difference were significant (p = 0.046 and p = 0.017), respectively. Whereas between the middle and high educational groups the difference was not statistically significant (p = 1.000). Regardless of from the risk assessment method there were no significant differences in the changes between the educational groups. Within the groups the CVD risk score was lower at the follow-up visit than at the baseline in all groups. The change was significant in the medium education group and in the high educational group. Although the Framingham risk score was higher at the follow-up visits than at baseline visit in all educational groups, the change was not statistically significant.

**Table 3:** Risk scores and change of the values between visits.

	Educational attainment					
	Low	Middle	High	P value^a^	All participants	P value^b^
	n = 31	n = 67	n = 87		n = 200	
CVD risk score						
Baseline	7.1 (1.7)	6.3(1.6)	5,8 (1.1)	0.001	6.2 (1.5)	
Follow-up	6.6 (2.5)	5.7 (2.2)	4.8 (1.7)	<0.001	5.5 (2.1)	
Change	−0.5 (2.3)	−0.6 (1.8)	−1.0 (1.7)	0.055	−0.7 (1.8)	<0.001
95% CI	−1.3 to 0.4	−1.0 to −0.2	−1.5 to −0.6		−1.0 to −0.5	
Framingham risk score						
Baseline	10.3 (2.5)	9.3 (2.7)	9.5 (2.3)	0.174	9.6 (2.6)	
Follow-up	11.2 (2.8)	9.8 (2.7)	9.5 (2.6)	0.018	10.0 (2.8)	
Change	0.9 (2.6)	0.5 (2.5)	0.0 (2.1)	0.063	0,4 (2.3)	0.036
95% CI	−0.1 to 1.8	−0.1 to 1.0	−0.5 to 0.6		0.0 to 0,7	

Data are means (SD),

a P values are for test of equality between educational groups,

b P values are for test of paired difference.

## Discussion

Socio-economical status is strongly and inversely associated with several health outcomes including CVD risk. In the present study, we observed that higher educational attainment was associated with less smoking, higher levels of physical activity and lower use of dairy products. Physical activity and intake of dairy products were positively influenced by health counselling in all socio-economic groups. Smoking was significantly reduced only in the highest educational attainment group. Observed changes in lifestyle factors influenced only little the traditional risk factors assessed. However, counselling had a marked influence on cardiovascular risk score, except in the lowest educational attainment group. Smoking is known to be strongly associated with low educational attainment and the likelihood of quitting smoking is lower in those with low educational attainment [13], [14]. The prevalence of smoking, including both daily and occasional smokers, among men aged 35 to 44 years in Finland was in 2006 31,3% and in 2008 36,3%, respectively [15], [16]. When comparing the study population with Finnish male smokers the prevalence was considerably higher in the present study. Those with the highest educational attainment showed the best ability to quit smoking. However, the reduction was only 8.9% (95% CI 1.9% to 16%) from baseline and the prevalence of smoking at follow-up was still over 40%. We must keep in mind that the aim of the intervention in present study was a more comprehensive change in overall life style not only targeting smoking. Physical activity is known to be associated with lower risk of CVD and type 2 diabetes [17], [18]. In general physical activity increased in all SES groups. The biggest increases were noted in the two lowest SES groups. The general national goal for physical activity at the time of the study was moderate to vigorous physical activity at least 90 minutes per week. In all SES groups one fourth of the study participants reached this goal. In the general population among men of similar age 55% in 2006 and 60% in 2008 were practising moderate to vigorous physical activity at least 2 to 3 times per week [15], [16]. Marked differences in the use of unsaturated fat were observed between the groups according to their educational attainment with very low use of unsaturated fat among those in the lowest educational group. The intervention had a marked influence on use of unsaturated fat in all groups. At follow-up half of the participants reported using unsaturated fats while at baseline users of unsaturated fat were less than a quarter. In the general population in Finland among men aged 35 to 44 years 63% in 2006 and 64.7% in 2008 use margarine on bread and 48.4% in 2006 and 51.3% in 2008 use vegetables oils for cooking, respectively [15], [16]. The study participants belonged to a high risk group according to the study design and therefore their baseline values for smoking were higher, physical activity and use of soft fat were lower than those observed in the population in general. This offers an excellent starting point for a lifestyle intervention. The overall influence of the intervention was positive in all educational groups. We observed that these men changed their lifestyle into a positive direction after health counselling. However when focusing upon traditional cardiovascular risk factors, i.e. obesity, blood pressure, lipids, and glucose concentrations only minor changes were observed. All cholesterol levels decreased during follow-up, which is in accordance with previous studies [19], [20], [21]. It has been shown that when saturated fat is replaced with unsaturated fat all cholesterols subclasses will decrease. Fasting glucose concentrations tended to increase slightly which can be interpreted as the normal increase in fasting glucose observed in association with increased age [22], [23]. Cardiovascular risk algorithms are routinely used to guide clinical decision making in many settings as recommended in European guidelines on cardiovascular disease prevention in clinical practice [24]. We observed that the lifestyle counselling had significant influence on CVD risk score especially in the two highest educational attainment groups. A similar trend was also noted in the lowest educational attainment group, but mainly due to lack of power did not reach statistical significance. Interestingly the Framingham risk score did not improve and on the contrary even higher risk scores were seen after the intervention. This is probably due to the fact that the Framingham risk score does not include physical activity and has only two categories of smoking, whereas the CVD risk score has nine categories of smoking. This makes the CVD risk score more sensitive to identify even small changes in lifestyle.

The main weakness of this study was the relatively small study sample size. As we studied only Finnish men aged 40 years it is not possible to generalise the results into other age-group or into women. We did not include a control group since the study participants were high risk individuals. For ethical reasons, it would have been irresponsible to refuse them health counselling. However, we compared the lifestyle changes of all participants with data from the literature (“Health Behaviour and Health among the Finnish Adult Population” our references 15 and 16).

In conclusion, the present study showed that lifestyle counselling is feasible in high risk men and lifestyle intervention works in all educational groups. Interestingly the biomedical risk factors, e.g. blood pressure and lipid assessed did not show improvements. From a practical point of view our findings stress the importance of using risk score calculators in health counselling instead of looking at individual risk factors.
